# Real-World Efficiency of Pharmacogenetic Screening for Carbamazepine-Induced Severe Cutaneous Adverse Reactions

**DOI:** 10.1371/journal.pone.0096990

**Published:** 2014-05-07

**Authors:** Zhibin Chen, Danny Liew, Patrick Kwan

**Affiliations:** 1 Department of Medicine, The University of Melbourne, Parkville, Australia; 2 Melbourne Brain Centre, Royal Melbourne Hospital, Parkville, Australia; 3 Melbourne EpiCentre, Royal Melbourne Hospital, Parkville, Australia; 4 Department of Neurology, Royal Melbourne Hospital, Parkville, Australia; 5 Department of Medicine and Therapeutics, Chinese University of Hong Kong, Prince of Wales Hospital, Hong Kong, China; Osaka University Graduate School of Medicine, Japan

## Abstract

**Objectives:**

We evaluated the cost and efficiency of routine HLA-B*15∶02 screening to prevent carbamazepine-induced Stevens-Johnson syndrome and toxic epidermal necrolysis (CBZ-SJS/TEN) in Hong Kong.

**Methods:**

Data were extracted from patients who commenced CBZ as the first-ever AED treatment or tested for HLA-B*15∶02 allele in three years before policy implementation (pre-policy: 16 September 2005 to 15 September 2008) and three years after (post-policy: 16 September 2008 to 15 September 2011). Using published unit costs, we estimated the cost of screening by comparing the costs to prevent and treat CBZ-SJS/TEN. We compared the number of person-tests needed and the cost to prevent resultant death with cancer screening programs.

**Results:**

The number of screening tests needed to prevent one case of CBZ-SJS/TEN was 442, and to prevent one resultant death was 1,474 to 8,840. The screening cost was $332 per person, of which 42% was attributed to an additional consultation to review result and prescribe appropriate medication. HLA-B*15∶02 screening expended $146,749 to prevent a case of CBZ-SJS/TEN, and $489,386– $2,934,986 to prevent a resultant death. The corresponding numbers of tests and costs for mammography and Pap smear to prevent death due to breast and cervical cancers were 7,150 and 7,000, and $614,900 and $273,000, respectively. Comparing to the SJS/TEN treatment cost, HLA-B*15∶02 screening would become cost saving if a point-of-care test of less than $37 was available.

**Conclusions:**

HLA-B*15∶02 screening is as efficient as mammography and Pap smear in preventing death. Development of point-of-care testing will vastly improve efficiency.

## Introduction

Carbamazepine (CBZ) is a first-line treatment for focal epilepsy [1]. It is also commonly prescribed for neuropathic pain and bipolar affective disorder. Stevens-Johnson syndrome (SJS) and toxic epidermal necrolysis (TEN) are rare but severe idiosyncratic cutaneous reactions that may be induced by CBZ in the initial phase of the treatment [2], [3]. Mortality rates are up to 5% in SJS and 30% in TEN [4]. A very strong association between the human leukocyte antigen (HLA) allele HLA-B*15∶02 and SJS/TEN induced by CBZ (CBZ-SJS/TEN) is now well known, especially among Han Chinese and South East Asians, of whom up to 15% are genetic carriers [5]–[9]. Screening for HLA-B*15∶02 in individuals with such ancestry prior to commencing CBZ, with avoidance of the drug in genetic carriers, was reported to be effective in preventing the disease [10]. This practice is recommended by drug regulatory agencies, such as the US Food and Drug Administration [11] and UK Medicines and Healthcare products Regulatory Agency [12], and is supported by international practice guidelines [13].

On 16 September 2008, a system-wide routine HLA-B*15∶02 screening policy was implemented in all public hospitals and clinics in Hong Kong. In the present study, we aimed to evaluate the efficiency of the screening policy in terms of the cost to prevent a case of CBZ-SJS/TEN and the resulting mortality, using observed data from routine clinical practice.

## Methods

Data were extracted from the Hong Kong Hospital Authority’s (HA) Clinical Data Repository, which contains the complete electronic medical records of all its patients dating back to 1994 [14]. The HA manages all public hospitals and outpatient clinics in Hong Kong [15], providing healthcare to over 90% of the 7 million residents, 93.6% of whom are Han Chinese and 5.2% other Asians [16]. The HA database integrates all the medical information of any given individual under a single portal, including diagnoses, inpatient discharge summaries, outpatient notes, information on medications dispensed, history of drug allergies and investigation results. Each patient has a unique identifier and data from all public hospitals and clinics throughout the city are stored in the database.

HLA-B*15∶02 carrier status was determined by sequence-based typing in National Association of Testing Authorities (NATA)-accredited pathology laboratories in Hong Kong’s two university hospitals [17]. This high resolution HLA typing platform is considered to be the conventional “gold standard”, hence assumed to be 100% sensitive and 100% specific in detecting carriers.

The study period covered three years before the implementation date (pre-policy: 16 September 2005 to 15 September 2008) and three years after (post-policy: 16 September 2008 to 15 September 2011). Patients of interest were those who had newly commenced CBZ as the first-ever AED or who were tested for the HLA-B*15∶02 allele. CBZ treatment was considered the first AED when there was no record of any AED prescription and allergy associated with AED in the previous 12 months or more (necessitating review of data from 16 September 2004).

SJS/TEN was attributed to CBZ if the patient was hospitalized for SJS/TEN within 90 days of commencing the drug and records did not suggest an alternative cause [4]. Identification of patients with SJS/TEN was based on discharge diagnostic codes (International Classification of Diseases, Ninth Revision, Clinical Modification [ICD-9-CM]), as defined by HA’s in-house clinical vocabulary and structured disease documentation system (the Clinical Data Framework), which has proven accuracy [18]. Further verification of the coding was performed in a random 10% sample of the patients by examining discharge summaries and relevant information including skin biopsies. All the sampled patients were confirmed to have SJS/TEN.

SJS/TEN treatment costs were derived from length of hospital stays observed for patients with CBZ-SJS/TEN (mean 23.8, standard deviation 22.8 days, Table S1). Costs of hospitalization per day, HLA-B*15∶02 testing, and outpatient consultations were based on published estimates [17], [19]. Based on the above, the cost of hospitalization for one episode of CBZ-SJS/TEN was assumed to be $16,441. Because the median result turnaround time for HLA-B*15∶02 testing was four days (interquartile range 3 to 7 days), and 98.6% (4,125/4,183) was more than one day, the costs of screening included an additional outpatient consultation. All costs were converted from Hong Kong (HK$) to US dollars ($) using the fixed exchange rate of HK$7.8 to $1 [20].

The prevalence of HLA-B*15∶02 was estimated form individuals tested without or before the development of AED-induced SJS/TEN. SJS/TEN incidence among CBZ recipients were summarized for the pre- and post-policy periods. The specific risks of CBZ-SJS/TEN among HLA-B*15∶02 carriers and non-carriers were extrapolated from observed HLA-B*15∶02 prevalence, risk of CBZ-SJS/TEN in the total population (comprising carriers and non-carriers) and reported odds ratio for CBZ-SJS/TEN conferred by a positive HLA-B*15∶02 status [21] (see Formulae 1–3 in Appendix S1).

The OR for the association between CBZ-SJS/TEN and presence of HLA-B*15∶02 in Hong Kong Han Chinese was reported to be 89.25 (95% confidence interval [CI] 19.25–413.83) [21]. For a rare condition like SJS/TEN, the OR would be a close approximation of the relative risk [4], [6]–[8], [21], [22].

To evaluate the efficiency of the pharmacogenetic screening, we compared the number of person-tests needed and the cost to prevent resultant death with two conventional cancer screening programs, mammography and pap smear. These cancer screening programs are broadly practiced and recommended worldwide [23]. To ensure the consistency in calculating the cost to prevent resultant death, only the costs of routine screening for the two cancer screening programs were considered, so the costs of confirmatory diagnostic testing, biopsy and all subsequent procedures were not included.

All analyses were performed using statistics software package *STATA 12* (StataCorp, College Station, TX).

### Ethics

The study was approved by the joint research ethics committee of the Chinese University of Hong Kong and HA’s New Territories East Cluster. Patient information was anonymized and de-identified prior to analysis.

## Results

A total of 4,136 individuals were tested in the post-policy period, of whom 4,124 did not have history of AED-induced SJS/TEN prior to testing. Among these individuals, 16.8% (691/4,124) were HLA-B*15∶02 positive. Therefore, as the reciprocal of the HLA-B*15∶02 prevalence, approximately six individuals are needed to screen to detect one HLA-B*15∶02 carrier in Hong Kong.

Among the 8,284 patients commenced on CBZ as the first-ever AED in the pre-policy period, 20 (0.24%) developed SJS/TEN, of whom one (5%) died (see demographic details for CBZ users in Table S2). None of the patients commenced on CBZ in the post-policy period (n = 1,076) developed SJS/TEN. The risk of SJS/TEN among all patients commenced on CBZ (0.24%) combined with the prevalence of HLA-B*15∶02 (16.8%) and OR for HLA-B*15∶02-associated CBZ-SJS/TEN (89.25) allowed for estimation of risks of CBZ-SJS/TEN specifically among HLA-B*15∶02 carriers and non-carriers (see Formula 3 in Appendix S1). These were 1.36% (95% CI 1.15%–1.42%) and 0.02% (95% CI 0.06%–0.003%), respectively.

Since the screening policy prevented CBZ-SJS/TEN in HLA-B*15∶02 carriers in the post-policy period and six individuals were needed to be screened to detect one genetic carrier, as the quotient of the number needed to screen to detect one biomarker carrier divided by the CBZ-SJS/TEN risk among HLA-B*15∶02 carriers, 442 individuals would need to be screened to prevent one case of CBZ-induced SJS/TEN (see Formula 5 in Appendix S1).

Based on the observed case fatality of 5%, the number of screening tests needed to prevent one death from CBZ-SJS/TEN would be 8,840 (see Formula 6 in Appendix S1). However, the observed case fatality was based on just a sample of one death. The literature reports case fatalities of SJS/TEN of up to 30% [24], which would equate to just 1,474 screening tests. By comparison, the numbers of screenings needed for mammography and Pap smears to prevent one death due to breast and cervical cancer are 7,150 and 7,000, respectively [25], [26] (Table 1).

**Table 1:** Number of person-tests of HLA-B*15∶02 screening compared to mammography and Pap smear.

	Total number of person-tests (95% CI)
HLA-B*15∶02 screening	
To detect a carrier of HLA-B*15∶02	6
To prevent a case of CBZ-SJS/TEN	442 (423–522)
To avoid death of a person from CBZ-SJS/TEN^1^	1,474–8,840
***Mammography***	
To avoid death of a person from breast cancer^2^	7,150
***Pap smear***	
To avoid death of a person from cervical cancer^3^	7,000

1 Overall SJS/TEN mortality rate ranged from 5% to 30%.

2 Based on the recommended biennial screening for 20 years from aged 50 years and detection of one case per 715 persons screened over this period. [25] Number excluded 20% non-compliance rate that was assumed in the original report.

3 Based on the recommended quinquennial screening for 35 years from aged 24 years and detection of one case per 1000 persons screened over this period. [26].

Based on the number of screening tests and the current HLA-B*15∶02 screening cost of $334 ($142 [43%] due to additional consultation) per person, we estimated it cost $146,749 to prevent one case of CBZ-SJS/TEN, and $489,386 to $2,934,986 to prevent one death due to CBZ-SJS/TEN (Table 2). If the screening test result could be made available rapidly so that an extra outpatient consultation would not be needed, the cost to prevent one CBZ-SJS/TEN case would be reduced to $84,864 (Table 2). The policy could become cost saving (i.e. cheaper than treating a case of SJS/TEN) if the cost of the rapid test was below $37.

**Table 2:** Cost of HLA-B*15∶02 screening compared with mammography and Pap smear.

	HLA-B*15∶02 screening^1^		Mammography†	Pap smear^2^ †
	Lower-limit	Upper-limit		
***Costs($)***				
Per lab test/screening	192		86	39
Per additional consultation	142			
SJS/TEN treatment	16,441			
***To prevent one indicated disease case***				
Laboratory test costs	84,864			
Additional consultation#	61,885			
Total	146,749			
***To avoid death from indicated disease***				
Laboratory test costs	283,008	1,697,280	614,900	273,000
Additional consultation#	206,378	1,237,706		
Total	489,386	2,934,986		

1 Upper-limit cost of HLA-B*15∶02 screening to prevent one death was estimated from overall SJS/TEN mortality rate at 5%, and 30% mortality rate was used for lower-limit.

2 Costs were converted from UK sterling to US dollars using the May 2004 exchange rate of £0.56 to $1.

† Excluded costs of diagnostic screening, biopsy and all subsequent procedures if any abnormality was found.

# Cost for an additional consultation was included based on the observed 98.6% non-same-day test turnaround rate.

SJS, Stevens-Johnson Syndrome; TEN, toxic epidermal necrolysis.

## Discussion

Recently, studies from Singapore and Thailand reported favorable efficiency and cost-effectiveness results for HLA-B*15∶02 screening prior to CBZ use [27]–[29]. However, these results were based on mathematical modelling and the data were not derived from population with active pharmacogenetic screening policy. Further, the prevalence of HLA-B*15∶02 and the costs associated with pharmacogenetic screening and health-care in these countries are considerable different from Hong Kong. These considerations prompted us to use real-world population data to quantify the efficiency of HLA-B*15∶02 screening to prevent CBZ-SJS/TEN in the Hong Kong setting.

Compared with other widely accepted screening tests for which periodic testing is needed, such as mammography and Pap smear for breast and cervical cancer, respectively, pharmacogenetic tests have the advantage of only needing to be undertaken once. In this regard, the number of screening tests required to prevent one death due to CBZ-SJS/TEN is broadly comparable to these cancer screening programs.

Compared to the direct screening costs of mammography and Pap smear (i.e. excluding confirmatory diagnosis and all subsequent treatment costs if abnormalities were found [30], [31]), the cost of the current HLA-B*15∶02 screening was similar and up to five times higher in preventing death, reflecting the variability in the severity, and hence mortality, of SJS/TEN (Table 2). The screening policy would be as cost-efficient as mammography and Pap smear if the overall mortality rate from SJS/TEN was higher than 11.5% and 28.3%, respectively. It is important to note that under the current practice, the screening cost was inflated owing to the additional cost associated with the long test turnaround time, necessitating an extra clinical consultation to review test results. Technologies (such as point-of-care testing [32]) which shorten the test turnaround time, ideally to same-day, could reduce the overall screening costs significantly, and could even lead to cost savings.

Although SJS/TEN is very different clinically and pathologically to breast and cervical cancer, all three conditions are similar in that they are serious, often-lethal diseases for which early interventions exist. Screening for breast and cervical cancer are universally recognized health strategies and as a consequence, often publicly funded. This is not true of screening for HLA-B*15∶02, being still quite novel. The intention of our study is to highlight that screening for HLA-B*15∶02 has the potential to be a worthwhile health strategy like screening for breast and cervical cancer, with comparable efficiency and cost-efficiency.

Our study only estimated the direct monetary effect of the HLA-B*15∶02 screening policy and did not assess potential health benefits from routine screening for the allele. Studies reported more than half of the TEN survivors suffered various serious long-term complications [33], including blindness [34]. In addition, there may be medico-legal consequence if testing recommendations from regulatory agencies are not followed. Compensation for TEN resulting from malpractice up to $63 million has been reported [35].

In conclusion, our study quantified the efficiency of the HLA-B*15∶02 screening policy in Hong Kong using observed real-world data. The results demonstrated that a pharmacogenetic screening policy could be as efficient as widely accepted cancer screening practices. However, development of point-of-care testing or other technologies to shorten the turnaround time is needed to improve the cost-efficiency.

## Supporting Information

Table S1 **Average length of stay and costs for carbamazepine-induced Stevens-Johnson syndrome and toxic epidermal necrolysis.**
(DOCX)

Table S2 **Demographics of patients received carbamazepine as the first-ever antiepileptic drug.**
(DOCX)

Appendix S1 **Formulae.** Formulae used for calculating the risks of CBZ-SJS/TEN among HLA-B*15∶02 carriers and non-carriers, and the numbers of patients needed to screen to prevent one CBZ-SJS/TEN case and death.
(DOCX)
