# Supplementary material for: Real-World Efficiency of Pharmacogenetic Screening for Carbamazepine-Induced Severe Cutaneous Adverse Reactions
Source: PLoS One. 2014 May 7;9(5):e96990. doi: 10.1371/journal.pone.0096990 (PMC4013087; doi:10.1371/journal.pone.0096990)
Supplement: Table S1 — Average length of stay and costs for carbamazepine-induced Stevens-Johnson syndrome and toxic epidermal necrolysis. (DOCX) [file pone.0096990.s001.docx]

| **Table S1. Average length of stay and costs for carbamazepine-induced Stevens-Johnson syndrome and toxic epidermal necrolysis** | | | | |
| --- | --- | --- | --- | --- |
|  | Average length of stay in days (S.D.) | | Daily Cost ($) [[19](#_ENREF_19)] | Overall Cost ($) |
| General ward/burns unit^1^ | 22.7 | (22.3) | 600 | 13,620 |
| ICU | 0.8 | (2.5) | 2,949 | 2,359 |
| HDU | 0.3 | (1.1) | 1,538 | 462 |
| Total SJS/TEN treatment | 23.8 | (22.8) | 691 | 16,441 |
| 1. Daily cost for burns unit was assumed to be the same as general ward. ICU, intensive care unit; HDU, high dependency unit | | | | |
