# Supplementary material for: Real-World Efficiency of Pharmacogenetic Screening for Carbamazepine-Induced Severe Cutaneous Adverse Reactions
Source: PLoS One. 2014 May 7;9(5):e96990. doi: 10.1371/journal.pone.0096990 (PMC4013087; doi:10.1371/journal.pone.0096990)
Supplement: Table S2 — Demographics of patients received carbamazepine as the first-ever antiepileptic drug. (DOCX) [file pone.0096990.s002.docx]

| **Table S2. Demographics of patients received carbamazepine as the first-ever antiepileptic drug** | | | | |
| --- | --- | --- | --- | --- |
|  | **Pre-policy**  **(16 Sep 2005 to 15 Sep 2008 )** | | **Post-policy**  **(16 Sep 2008 to 15 Sep 2011 )** | |
| ***Patients used CBZ as initial AED treatment*** | 8,284 | | 1,076 | |
|  |  |  |  |  |
| ***Inpatients (%)*** | 6,455 | (78%) | 790 | (73%) |
| Males (% of inpatients) | 3,074 | (48%) | 367 | (46%) |
| Median age (IQR) | 60 | (45 - 74) | 50.5 | (28 - 70) |
| Epilepsy or seizures (% of inpatients) | 761 | (12%) | 230 | (29%) |
| Other indications (% of inpatients) | 5,694 | (88%) | 560 | (71%) |
|  |  |  |  |  |
| ***Patients developed CBZ-SJS/TEN (%)*** | 20 | (0.24%) | 0 | (0%) |
| Fatal cases (% of CBZ-SJS/TEN) | 1 | (5%) | 0 | (0%) |
| AED, antiepileptic drug; CBZ, carbamazepine; IQR, interquartile range; SJS, Stevens-Johnson syndrome; TEN, toxic epidermal necrolysis | | | | |
