# Supplementary material for: Real-World Efficiency of Pharmacogenetic Screening for Carbamazepine-Induced Severe Cutaneous Adverse Reactions
Source: PLoS One. 2014 May 7;9(5):e96990. doi: 10.1371/journal.pone.0096990 (PMC4013087; doi:10.1371/journal.pone.0096990)
Supplement: Appendix S1 — Formulae. Formulae used for calculating the risks of CBZ-SJS/TEN among HLA-B*15∶02 carriers and non-carriers, and the numbers of patients needed to screen to prevent one CBZ-SJS/TEN case and death. (DOCX) [file pone.0096990.s003.docx]

# APPENDIX S1

## Formulae

Formulae for calculating risks of CBZ-SJS/TEN among HLA-B*15:02 carriers and non-carriers:

$R_{c}=OR\times R_{o}$ (1)

$p\times R_{c}+\left(1-p\right)\times R_{o}=R_{t}$ (2)

combining Formulae 1 and 2:

$$p\times OR\times R_{o}+(1-p)\times R_{o}=R_{t}$$

$$R_{o}\times\left[\left(OR\times p\right)+(1-p)\right]=R_{t}$$

$R_{o}=\frac{R_{t}}{\left(OR\times p\right)+\left(1-p\right)}$ (3)

where: R_c_ = risk among HLA-B*15:02 carriers, R_o_ = risk among non-carriers, R_t_ = risk in the total population, OR = odds ratio for CBZ-SJS/TEN conferred by a positive HLA-B*15:02 status, and p = HLA-B*15:02 prevalence in the population.

Formulae for calculating numbers of patients needed to screen to prevent one CBZ-SJS/TEN case and death:

$NNS_{HLA-B*15:02}=\frac{1}{p}$ (4)

$NNS_{case}=\frac{NNS_{HLA-B*15:02}}{R_{\Delta}}$ (5)

$NNS_{death}=\frac{NNS_{case}}{R_{M}}$ (6)

where: NNS_HLA-B*15:02_ = number of patients needed to be screened to detect one HLA-B*15:02 carrier, NNS_case_ = number of patients needed to be screened to prevent one CBZ-SJS/TEN case, NNS_death_ = number of patients needed to be screened to prevent one death from CBZ-SJS/TEN, R_Δ_ = change of risks among HLA-B*15:02 carriers between the pre and post-policy periods, and R_M_ = mortality rate of CBZ-SJS/TEN, and p = HLA-B*15:02 prevalence in the population.
